# The Influence of Sensory Characteristics of Game Meat on Consumer Neuroperception: A Narrative Review

**DOI:** 10.3390/foods12061341

**Published:** 2023-03-22

**Authors:** Marius-Mihai Ciobanu, Diana-Remina Manoliu, Mihai-Cătălin Ciobotaru, Bianca-Georgiana Anchidin, Mădălina Matei, Mugurel Munteanu, Gabriela Frunză, Otilia Cristina Murariu, Elena-Iuliana Flocea, Paul-Corneliu Boișteanu

**Affiliations:** 1 Faculty of Agriculture, “Ion Ionescu de la Brad” University of Life Sciences, M. Sadoveanu Alley, No. 3, 700490 Iasi, Romania; 2 Faculty of Animal and Food Resources Engineering, “Ion Ionescu de la Brad” University of Life Sciences, M. Sadoveanu Alley, No. 8, 700490 Iasi, Romania

**Keywords:** game meat, game meat products, sensory properties, neuroperception, consumer behavior, consumer perception, biological processes

## Abstract

Game meat contains bioactive compounds that directly influence the formation of a rich reservoir of flavor precursors that produce specific sensory properties. Quality is considered one of the most influential determinants of consumer behavior, but the interpretation of this concept differs between consumers. Although recognized for its quality, its unique sensory characteristics (smell, taste, aroma) may have a major impact on consumer perception. The aim of this review is to describe the consumer behavior regarding game meat through elements of neuroperception, using methods of analysis, observation, and interpretation of scientific information from the literature. Following the analysis of published papers on this topic, it was shown that external factors influencing the biological basis of behavior could provide explanations for the acceptance or rejection of this type of meat and solutions. Neuroperception can explain the mechanism behind consumer decision-making. The influence of extrinsic factors (environment, mood, emotions, stress) shapes the perception of the quality attributes of game meat, the unique sensory characteristics of game meat passing through a primary filter of sensory receptors (eyes, nose, tongue, etc). Game meat is darker and tougher (compared to meat from domestic animals), and the taste and smell have the power to trigger memories and change the mood, influencing consumer behavior. Understanding consumer attitudes towards game meat in relation to quality attributes and the physiology of sensory perception can provide important insights for food industry professionals, processors, sensory evaluators, and researchers.

## 1. Introduction

In the last 15 years, consumer neuroscience has become a topic of increased interest for various fields economics, marketing, psychology [1,2,3,4] of food industry researchers [5,6,7]. According to research, neuroscience can help researchers, sensory evaluators, and manufacturers in the food industry or may contribute to understanding consumer response which has a high degree of subjectivity about products due to simplistic understanding and examination of food without control over the actual judgmental capacity of the sense organs and the objectivity of the analyst’s reasoning [5]. In a recent study, Yoon et al. [4] argue that neuroscience suggests new hypotheses regarding choices and underlying pathways that are consistent with our understanding of the biological processes and permit the use of neural data in order to have a precise prediction about consumer behavior.

In order to better understand the complexity of the brain and how it influences behavior, data about molecular biology need to be considered. Various researchers [8,9] have stated that dopamine is a neurotransmitter that plays an important role in human behavior, being released when a sense of safety is mastered. Rewarding stimuli such as food and water are linked to the effectiveness of dopamine release in the *nucleus accumbens*. Moreover, dopamine release in a wider range of structures is involved in memory loading.

Studies have demonstrated that neurons expressing oxytocin receptor (OTR) are a specific class of glutamate neurons that project mainly to the central component of the *nucleus accumbens* and to posterior amygdala. Along with anterograde neural tracing, photo-stimulation of projections from the paraventricular thalamus of the *nucleus accumbens* (PVT-NAcC) is signaling the food searching, while a judicious activation of projections to the posterior basolateral amygdala (PVT-pBLA) is resulting in a reduced effect on feeding [10]. Therefore, it was concluded that neurons expressing ORT in the paraventricular thalamus and their projections to the *nucleus accumbens* are responsible for the regulation of a healthy feeding motivation [10]. The olfactory mechanism combines the smell data requested for generating an appropriate behavioral outcome. He et al. [11] suggested that a certain pathway is activated, involving a serotonin signaling way, which may selectively control the behavioral reply to the main olfactory stimuli. Under different conditions, including stress, injuries, or various infections, neurocircuits might be activated via peptide-containing neurons. These neurons are known to be linked to the calcitonin gene in the parabrachial nucleus, which functions as a “switch” for feeding [7].

Although quality, a concept that is widely used nowadays, is perceived as one of the most crucial factors that regulates consumer behavior, the word is interpreted differently by consumers [12,13,14,15,16,17]. Accordingly, a growing number of consumers are consciously addressing the issue of ethical or emotional food sourcing [18,19,20,21]. In meat consumption, factors impacting consumer behavior also involve beliefs about the psycho-emotional understanding of the hunting activity, sensory traits, nutritional and dietary value [19].

Various studies have proved the notable nutritional value of game meat, including the high protein content, low fat, a satisfactory composition of n3–n6 polyunsaturated fatty acids and minerals [22,23]. Game meat is a source of bioactive contents, including conjugated linoleic acid (CLA), as well as different beneficial peptides such as anserine or carnosine [19]. Moreover, the aforementioned peptides and its analogs have been shown to be able to significantly reduce the incidence of heart attack and improve neuronal function [17,24,25,26,27,28,29].

While some researchers explore the sensory features, the remarkable quality of game meat, or the theory of game meat sustainability in the alimentary sphere [30,31,32,33,34], others have discovered that consumers are not a homogeneous unit, and their behavior remains far from clear [35]. Indeed, Yoon et al. [4] suggested that neuroscience could be able to provide new explanations regarding the multiple sources of heterogeneity observed between populations. Therefore, the consumer consumption choice behavior in relation to the corresponding importance of game meat might be a significant domain of research. Consumer choices have been proposed to be generated by various considerations recognized as permanent predispositions that direct human behavior. According to the effect resulted, two types of reasons have been described: emotional reasons (which are influenced by certain psycho-emotional factors) and rational reasons (which leads to satisfaction related to the consumption of food with specific properties and expected quality) [36,37,38].

Neural sensory systems, such as the visual, gustatory, and flavor-related structures, are responsible for the body’s response to sensory exposure to food [39]. The neuroperception generated by food, in particular game meat, involves the use of the following senses: visual, auditory, olfactory, and gustative. Visual cues are often the first perceived and able to influence perception [40]. People derive food expectations from the visual properties of products. Venison meat compared to farm animal meat is darker in color, the color being associated with a specific taste. In addition, color attracts attention, memory, and distracts individuals from observing the essential characteristics of the product. The smell is another factor that has the power to alter perception. The smell of game meat is specific, its impact on perception is explained by the familiarity of the individual with the smell [41].

The salivary and gastric response following exposure (visual and olfactory) to a food is related with the hedonic value of that product (with the pleasure of consumption). Sensory characteristics of food perceived visually, gustatorily, and olfactorily represent elements of nutritional recognition (e.g., amount of salt, fat), product condition (related to texture), or origin (e.g. via perceived volatiles) [39]. The textural perception of food is known to be initially interpreted through visual perception, then through extraoral physical contact, and finally within the oral cavity (via the tongue, oral membrane mucosa, periodontal ligament, receptors, and thermoreceptors) [42].

The olfactory system has direct connections with brain components involved in memory and emotion, including the hippocampus, thalamus, and frontal cortex [43]. The pattern in which emotions and food memory control the decision-making is explained by cognitive systems, which is well known to influence the response decision-making processes. When product flavor is related with a previous experienced representation, involving the element of familiarity at the same time, other components of the product are experienced in the oral cavity [44]. Cognitive neuroscience helps researchers explain cross-modal interaction and multisensory integration [45]. As stated by Beekman [40], environmental factors have a crucial impact on sensory perception. Various empirical evidences has shown that consumer perception and emotional responses differ according to environmental situations [46], e.g., ambient odor, table setting, temperature during tasting, or even season. In terms of future research, a methodology based on a series of scientific facts, in an appropriate environment, can be applied for the psycho-sensory evaluation of game meat products by customers.

Since the rejection of game meat by some consumers has been frequently reported, a better understanding of the reasons of this rejection was the point in the conception of this review [19,38,47,48]. Moreover, new insights may be provided by understanding consumer attitudes directed to the game meat in relation to quality characteristics and the physiology of sensory perception. External factors influencing the behavior of biological mechanisms could provide solid justifications for game meat rejection. In order to address these concerns, the main purpose of this review is to explain consumer behavior based on sensory characteristics and neuroperception. According to our purpose, different papers on this topic were reviewed to highlight the expression of agreement or refusal regarding game meat over the last two decades.

## 2. Materials and Methods

Several literature-based bibliographic sources were consulted in order to meet the study’s objectives. The database used for the literature research included PubMed Central, ScienceDirect, and Web of Science. During the database search, a number of keywords, including “neuroscience”, “neurotransmitters”, “molecular biology”, “venison”, “perception”, “preference”, “game meat”, “consumer behavior”, “consumer choice”, and phrases such as “game meat quality”, “consumers attitudes toward game meat”, and “sensory profile of game meat” were used. Based on the title, abstract, and complete content, all the relevant studies were selected. In addition, the research was supplemented by consulting some bibliographical sources in the Romanian language.

The research methods used in this study were analysis, observation, and interpretation of data from the literature on previous studies published in the last two decades on consumer behavior in accepting or rejecting game meat, as well as neural implications that might explain consumer behavior. In order to be included in our review, we chose studies retrieved by the scientific databases that were published over the last two decades. Five authors (E.-I.F.; M.M.^1^; D.-R.M.; B.-G.A.; M.-C.C.) individually examined the data, and any differences were resolved through debate and agreement with the other five authors (M.-M.C.; M.M.^2^; P.-C.B.; O.C.M.; G.F.).

## 3. Results and Discussion

### 3.1. The Trophic and Biological Importance of Game Meat

As part of natural ecosystems, humans have always relied on the earth’s natural resources for food to survive, while wildlife was always one of the most accessible resources [49].

In Europe, where mountain areas occupy a significant percentage of the existing sur-faces, the game species are considered an important resource of these mountain ecosystems [50]. Therefore, there is a vast diversity of the game species, including the roe deer (*Capreolus capreolus),* the fallow deer (*Dama dama* L.), or the wild boar (*Sus scrofa*), which in turn are widespread in ecosystems and, consecutively, the most hunted. A significant part of hunted animals is consisting in small game species including wild rabbits, pheasants, grouse, as well as a significant variety of sedentary or migratory birds [51].

Considering the biological aspect, the aforementioned species (roe deer, fallow deer, wild boar, rabbits) represent valuable animals due to their favorable nutritional content such as fatty acids [52,53,54], proteins with high biological value, essential amino acids, micronutrients or other compounds (carnitine, conjugated linoleic acid—CLA) [55,56].

The future food crisis due to the increase in global population is being discussed more and more. Meat, a basic pillar in human nutrition, challenges food industry specialists to find durable and healthy alternatives through the consumer perception of meat quality. Game meat could meet the needs of modern consumers, as an alternative to meat from farm animals, since different studies suggested a superior quality [52,53,56,57,58].

In perspective, the recent topics target uninformed consumers who, on an emotional basis, respond by rejecting game meat products and who, through appropriate education, can use these resources favorably.

### 3.2. The Economic Importance of Game Meat

Wildlife management has become a necessity in recent years in order to balance the needs of humans and nature. Ensuring sustainability in wildlife management helps to sustain fauna in the face of continuing global pressures on wildlife, particularly as a result of human population growth, urbanization, and changing lifestyles.

More and more studies are reporting a growing number of European wild animal populations. Abundant breeding of wild animals could cause major damage by replacing from their natural habitat. Thus, large wild animals (e.g.: wild boar, red deer, roe deer) can be a sustainable alternative to farm animals.

Wildlife management describes the concept of rational management of wild species to maintain populations and habitats over time, taking into account the socio-economic needs of people. If properly managed, game fauna can provide food and contribute considerably to the economic sustainability of the population, as well as to the protection of human health and the environment. However, situations of wildlife overabundance cause many species to be considered harmful to natural habitat, and the society’s tolerance for those species decreases, leading to a decline in interest in wildlife. The economic impact of ecological imbalances caused by the inappropriate wildlife management can be particularly high, which is why important measures for each type of damage are necessary in order to balance the economy [59].

From an applied perspective, the species of hunting interest contribute to a better livelihood and provide essential ecosystem services for the entire population, being at the same time resources that provide the direct use of the values held in form of meat, recreational hunting tourism, or hunting trophies, all with high economic value [60]. However, the overall financial value of wildlife species to society is prologued far beyond their direct use as a result of the hunting process, and may include indirect use values, choice values, and subsistence values [61].

According to Whitnall and Pitts [62], during the last two decades, meat consumption has registered an increase of 58%, reaching 360 million tons in 2018; interestingly, 54% of this augmentation was due to global population expansion, while the rest was attributed to an increase in consumption per capita, rolled by changes in consumer dietary preferences and income improvement. Over recent years, due to the need for sustainable use of resources, the advantages of game meat have been intensively popularized, although in many European countries, such as Sweden, Germany, Great Britain, Croatia, the Czech Republic, and Norway, the consumption is reduced. Therefore, the official range of game meat consumption of is low, from 0,2 to 1,1 kg meat/per person/per year, while only 2–4% of the population regularly consumes game meat [19]. The main reasons explaining the low consumption are the high price, lower availability (in comparison to the meat of domestic animals), the limited methods of culinary preparation, and the lack of information about the hunting sector.

From the socio-economic point of view, hunting is considered a special activity, notably in rural areas. According to some recent research reports, it was indicated that in the European Union (EU), hunting is worth approximately €16 billion [63]. For instance, in a case study, the economic value of an 84 kg red deer carcass was estimated, revealing that the total value of the carcass was calculated at €504.99, that is, €6.00/kg [59], a much higher value compared to the meat of some of the farm animals. The numerous hunting funds existing worldwide often ensure an important regional economic development, together with a fruitful development of local tourism. If managed sustainably, wildlife can provide food and contribute considerably to the economic sustainability of the population, as well as to the protection of human health and the environment [64,65].

Effective measures to reduce or eliminate damage caused by wild ungulates could also contribute to the ecological dimension of game management. In accordance with economic life, management measures centered on the game habitat, the management of vegetation and green areas, or the infrastructure adaptation measures might be useful to reduce the totality of the damage created by wild ungulates on the area in order to reduce conflicts with other human activities and improve the population’s tolerance towards these species and perception of this natural resource [66].

Such actions can be developed to strengthen the principles of sustainable management, while the use of resources by informing and involving stakeholders and the general public would be considered a joint effort and, moreover, a key to developing and improving the management of wildlife as a provider of food resources [67,68,69].

### 3.3. Game Meat Nutritional Quality

Based on the studies published in the recent years on behavioral elements of meat consumers, some trends were noted, such an increase in population nutritional aware-ness and the importance of the quality component provided by the consumption of game meat [19].

As a concept, quality is an interchangeable variable related to consumer expectations and needs a term that cannot be assigned a single and complete definition [13]. However, addressing quality-related topics always shows that this non-unique concept is considered one of the decisive factors in consumer behavior and decisions.

In describing the reasons for choosing game meat, quality attributes are the defining elements for the interest of consumers who have become more demanding and more pre-occupied with the food they eat. The trends of its consumption are increasing rapidly due to the intrinsic characteristics, given by the nutritional value and health benefits, and the extrinsic characteristics of game meat, given by the origin of the products and social-economic factors [19,70].

Thus, a detailed monitoring of nutritional properties and the contribution of sensory properties have thus become important elements to guarantee the quality of game meat obtained [71].

Accordingly, the high nutritional value provided by a rich protein content with high biological value and the beneficial composition of favorable amino acids and essential fatty acids were mentioned by several authors such as Blaska et al. [72], Quaresma et al. [73], Valencak et al. [23], and Frunză et al. [54]. Similarly, other studies found interesting associations between game meat and the concept of a healthy product due to the limited content of saturated fat [74]. Depending on age, physiological state, conditions or hunting season, different variations in game meat quality have been reported [23].

With respect to the nutritional value and sensory properties, venison meets the demanding expectations of specialists [75]. Accordingly, this aspect is well described in the literature; therefore, Table 1 shows the nutritional value of game meat from different species mostly preferred by consumers.

Depending on the species of wild animal, the protein content of game meat ranges between 17 and 26%. All of the essential amino acids, including isoleucine, leucine, lysine, methionine, cystine, phenylalanine, tyrosine, threonine, tryptophan, and valine, are found in these complete proteins. The highest proportion of essential amino acids were reported in wild boar muscles, approximately 7.99 g/100 g [91]. It is well documented that aromatic amino acids, including tyrosine, tryptophan and phenylalanine [92], are carried to the central nervous system (CNS) and are able to cross the blood–brain barrier [93]. Additionally, dopamine, norepinephrine, and epinephrine are synthesized sequentially from tyrosine [94,95,96].

According to different authors [57,77,85], research on the chemical composition of muscle tissues (m. *Logissimus lumborum*, *Longissimus dorsi*, *Semitendinosus*, and *Triceps brachii*) harvested from different game species (*Cervus elaphus* L., *Sus scrofa ferus* harvested from N-E Romania, *Cervus canadensis*, *Capreolus capreolus*, *Sus scrofa*, *Cervus capreolinae* harvested from Latvia) revealed the following composition: 74% water, 22% proteins, 2–3% lipids, and 1–2% other soluble non-protein substances. On analyzing the composition, it was confirmed that water is the main element of the muscles, with variable proportions according to the species, age, gender, diet, fattening status, or muscle group.

The meat main nutritional component is constituted by the dry matter, which represents approximately one-third of the composition, while the nutritional balance is conferred by the presence of macronutrients (proteins, lipids), chemical components that define the intrinsic quality of the meat, and by the minerals and vitamins, compounds with a major role in the body’s metabolic processes [97].

When compared to domestic farm animals, the game meat amino acid composition is not much different, revealing a balanced ratio of essential and non-essential amino acids. Thus, in the specialized literature, the superior value of game meat is confirmed by a higher content of some essential amino acids. Various studies reported comparative data regarding the higher values of the total proteins of game meat and of domestic farm species [77,98]. Accordingly, Okuskhanova et al. [76] revealed that maral meat has a higher content in essential amino acids compared to beef. Moreover, according to Bureš et al. [99], game meat of fallow deer and red deer contains a greater concentration of polyunsaturated fatty acid (PUFA), especially ω-3 PUFA and ω-6 PUFA, compared to beef meat. In order to sustain these data, the values of amino acids and fatty acids content of game meat from different species are presented in Table 2.

Kim et al. [103], in their research where they attempted to purify a novel antioxidant peptide (APVPH I) from venison, demonstrated that in the presence of the peptide, the levels of antioxidant enzymes in neuronal cells were increased. These neuroprotective peptides are of interest to researchers since their molecular characteristics underlie the well-being of the nervous system [104]. The presence of APVPH I downregulated the generation of NO (a free radical that leads to pathologic disorders), inhibited the generation of ROS (reactive oxygen species), decreased the lipid peroxidation by its hydrophobic property that allows it to donate protons to lipid-derived radicals as a result of the communication with lipid molecules [103,105].

Different studies reported certain mechanism such as the hydrolyzed deer polypeptides and polypeptides in velvet horns prevented oxidative stress and cell death by regulating the Bax/BBC-2 ratio [103,106]. Xia et al. [107] suggested that peptides extracted from deer antler can manifest a neuroprotective ability, with a positive effect on neurological diseases through their anti-inflammatory activity and antioxidant properties. The human body is capable of producing only nine amino acids (histidine, isoleucine, leucine, lysine, methionine, threonine, tryptophan, phenylalanine, valine) of the 22 amino acids. It is pointed out that a deficiency of one of the amino acids leads to the cessation of the synthesis of proteins and other necessary biological substances [76,108].

Integrated into the concept of quality, the safety of game meat consumption represents, similarly to the nutritional value, an important factor in terms for consumer perception of game meat [21].

The specific nutritional and sensory properties of game meat are given precisely by its natural origin and specific physiology [73]. Although minimal risks may exist regarding the consumption of game meat, according to studies in the literature mentioning risks related to the incidence of zoonoses [109,110,111], microbiological contamination [112] or different contaminants and pollutants [113,114,115], preventive measures are implemented to ensure the safety for consumption [111].

### 3.4. Sensory Characteristics of Game Meat

In addition to the nutritional properties, the consistency of game meat quality is given by its sensory properties. Given the complexity of the nutritional composition of game meat corelated to a sensory profile, the particular nutritional value provides the mix of characteristics that is resulting in overall food satisfaction [116,117]. The main particularities of game meat quality sensory assessment are consisting in the evolution of its gustatory satisfaction, in tenderness, juiciness, and aroma assessment [21]. The structural elements of the muscle fibers are the indicators that differentiate these properties, all of which are directly correlated with the body weights of the animals [118]. Meat structure contribute decisively to its sensory attributes. As a consequence of the changes undergone during thermal processing, the meat components constitute a reservoir rich in flavor precursors that produce specific characteristics. For instance, various molecules, such as the peptides and nucleotides directly influence the formation of volatile aromatic compounds. Similarly, lipids have an important influence on the flavor of meat since the volatile profile of aromatic compounds is generally dominated by lipid compounds [117].

The sensory quality of game meat is based mainly on its characteristics related to the appearance in the unprocessed condition, followed by the attributes identified after the preparation, such as texture, succulence, tenderness, taste, aroma, and attributes often described by instrumental methods or sensory testing. Game meat is darker, tougher, and tastes stronger compared to meat from domestic farm animals [119].

In general, unique sensory attributes possess a major influence on consumer perception. Consumer attitudes towards the game meat sensory characteristics differ according to their level of familiarity with the meat and their willingness to try new and unfamiliar products [21]. Various studies point out that an essential element that distinguishes meat of farm animals from game meat is consisting in flavor [21,120]. Taste has a major impact on consumer attitudes [38]. It was demonstrated that odor stimuli produce divergent perceptions in the human brain. Consequently, the basic deficiency of a human sensory response related to sensory susceptibility is resulting in low repeatability and reproducibility [121]. Some studies conducted on red deer meat, for example, have reported higher odor intensity compared to beef, a more intense flavor and aroma, properties that are accepted and appreciated by some consumers, while for others represents a major deterrent of consumption [21,99,122].

Moreover, Martin [123] brought to our attention that smell and taste are the most misunderstood senses. However, the sense of smell and taste has the power to evoke memories, change our mood, and even influence our behavior. It has also been shown that the synapses made by sensory neurons, which are responsible for transmitting all the information from the nose to the brain, can be modified by serotonin. Because serotonin-releasing neurons change their firing according to behavioral states, behavioral states can alter how odors are transmitted to the brain [124]. Sensory characteristics of game meat play an important role in consumers satisfaction, and their consistency remains imperative for consumer behavior [125].

### 3.5. Contribution of Sensory Characteristics and Psychological Considerations of Sensory Analysis

Considering the complexity of the characteristics of game meat, sensory attraction implies the characterization of the product through some complex perceptions, which in turn are associated with the psychology and psychophysics of human sensory perception and the evaluation of human behavior during the consumption [126].

Consumer behavior is a complex phenomenon defined by a series of factors that influence the human psyche, while the criteria for choosing products do not always correspond to real needs. In particular, the study of consumer attitudes and perceptions of game meat refers in particular to psychological factors that influence consumer decisions but also to environmental factors that include economic, social, cultural, or consumption context criteria [127].

**Figure 1:**
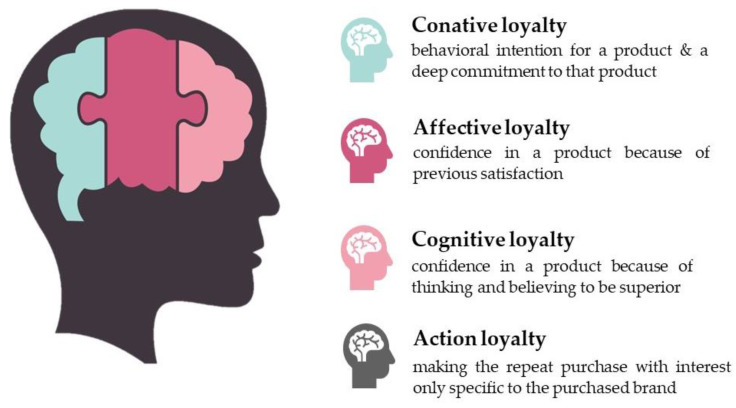
The behavioral and attitudinal elements of loyalty. Processing content according to Silayoi et al. [128]. Graphics: Copyright © 2010–2023 Freepik Company S.L. All rights reserved.

In the literature, the perception of the modern consumer has become a dual and complex concept consisting of the pre-purchase value of a product and the post-purchase value. As Silayoi et al. [128] noted, consumer behavior explained by neuroscience associated with the consumer satisfaction is resulting in the concept of loyalty as a general attitude towards a product. Indeed, consumer loyalty is mainly defined into two dimensions: the behavioral and attitudinal dimensions. The behavioral dimension refers to the frequency with which consumers repeatedly buy a product, while the attitudinal dimension takes into consideration the psychological commitment (decisional or evaluative) in accepting or rejecting the product [129]. The attitudinal dimension also includes consumers’ beliefs about a product, influenced by experience and level of knowledge. These aspects are important due to specific perceptions regarding product characteristics and sensory quality that influence consumers’ ulterior decisions [130]. The behavioral and attitudinal elements of loyalty are shown in Figure 1.

Essentially, loyalty is displayed through different stages consisting in (1) conative, an intention to purchase the product; (2) attitude, an affective preference for the product; and (3) belief, a preference for the product characteristics. Therefore, consumers become loyal to a product first in a conative sense, followed by an affective “liking” or “disliking,” and later in a cognitive sense [131]. Hence, consumer loyalty and commitment to the product are strengthened as each of the loyalty phases is overcome.

Between sensory analysis and the evaluation of its psychological impact on humans, the actual differences in perception between individuals constitute the part of the variability that sensory analysts want to monitor and psychological analysts want to quantify.

A complete perceptual experience appears by summing up all the component sensations caused by several factors such as sensations of an organoleptic nature, sensations and perceptions of an affective nature, or sensations associated with environmental conditions, all of which continuously interact to create the final acceptability. Examples that reflect the association of sensory evaluation with the psychological side of an individual have been outlined in various types of research studies where the interaction manner of product color with its taste and smell have been highlighted, revealing that positive evaluations of aroma are increasing as the color intensifies [132,133]. Font-i-Furnols and Guerrero [134] state that psychological factors such as cultural aspects, habits, and familiarity influence perceptions on the sensory quality. In the case of meat, consumers associate color with fresh meat characteristics, affirmation supported by consumers statements: red–purple color is associated with fresh meat, while the brown color of meat is perceived as an indicator of spoilage [134].

The psychological implications on the sensory evaluation of an alimentary product take into account the wide variety of psychological reasons underlying the determination of the act of consumption (sensory appeal of the product, nutrient content, price, functionality of the product, or various ethical reasons) [127].

These arguments are able to induce and direct consumer behavior to fulfill ex-pressed demands; specifically, these arguments can be rational, when they result in behavior appropriate to the context, and emotional, when they cause spontaneous actions followed by temporary emotional states. The vast majority of the reasons that direct consumer behavior with regard to a food product are emotional and may be associated with certain psychologically induced desires, embodied in the achievement of a certain level of psychological comfort which resulted following the consumption of specific foods [135].

Another important psychological factor that can influence eating behavior and the appreciation of a product refers to the individual’s attitudes towards that product, respective to the totality of the ideas associated with that food by the people. The relationship between food choice and emotional state is complex, with some foods being valued more only through the lens of improving mental state. Food preferences and the degree of appreciation for a certain product depend mostly on the emotional disposition of the individual, while the relationship between mental state and appetite being reflected more obviously among women [127].

**Figure 2:**
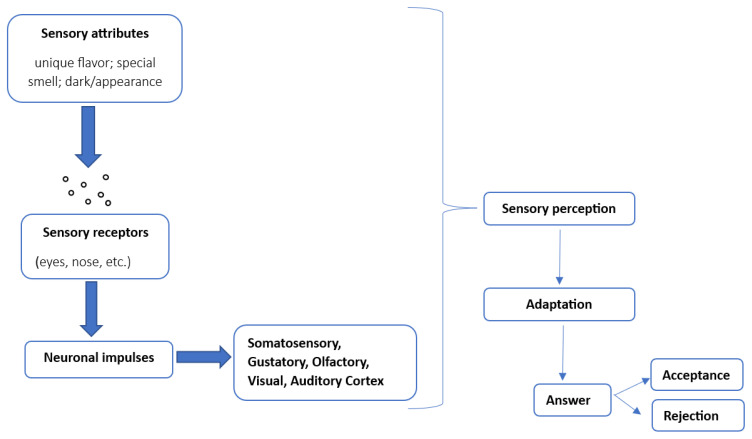
General approach of the influence of sensory attributes on sensory perception. Processing content according to Steele [136].

Ross [126] explained the relationship between sensory stimuli and human responses in relation to the physiology of sensory perception and how individuals perceive a product. A general approach of the influence of sensory attributes on the sensory perception that determine the consumer’s decision of acceptance or rejection is shown in Figure 2.

Stimuli are environmental factors that are causing sensory impressions or perceptions [126], while in order to transmit information to the brain via the CNS each sense organ activates a certain range of stimuli. At the level of the central nervous system, the sensory perception of a stimulus takes place through a chain of reactions. A stimulus generates a response through a nerve signal to the brain and its specific receptors interpret the information received through a perception that in turn will be translated into an individual response, a response that will be proportional with the intensity of the stimulus. Sensory perception is believed to depend on mixed interplay of neural circuits that process data in a cortical layer-mediated way, demanding specific feedforward/feedback loops generated by thalamocortical, corticothalamic corticocortical, and intracortical structures [137]. Sensory stimuli converted by sensory organs arrive to specific thalamic nuclei that disseminate information to primary sensory zones, which in turn screen and ultimately send information to secondary sensory zones [138,139]. Cortical adaptation is more than an information filter; lengthened contact to smell involves a chance to gain experience and familiarity with smell; therefore cortical adaptation is a necessary element of olfactory perceptual learning. Consequently, as an odor becomes more familiar, it becomes more distinct from other similar odors [140,141].

Sensory perception can generate objective responses that measure the intensity of the stimulus and the sensation. Based on the relationship between the physiological response of the CNS and the intrinsic reaction of the individual, subjective responses may provide results consisting in statements that people make about the sensations perceived [126]. For each sense organ, the stimulus is perceived differently by the central nervous system. Based on the knowledge, training, and acuity of each sense organ and communication skills, people may have sense perceptions, with interactions common across all five senses [142,143].

### 3.6. Consumer Neuroperception of Game Meat

Fear induced by certain misunderstood information activates a circuit responsible for the pathophysiology of neurodegenerative conditions. The secretion of serotonin (5-HT) in large amounts in the basolateral amygdala during fear memory consolidation sustain possible hypotheses for how 5-HT neurons encode disinterested stimuli and cues. The localization of specific 5-HT receptors is considered crucial in understanding the role of 5-HT in emotional behavior [144].

Product preferences were shown to be driven by cellular factors in the dorsal striatum, such that preferred food choices were activated when the mGluR3-AC5 pathway was inactive or mGluR1 was active. The AC5 (adenylyl cyclase type 5) system and mGluRs in the dorsal striatum are on/off molecular systems for direct food preference decisions for cue-guided options [145].

It is confirmed that dorsal striatal dopamine plays a role in the complexity of food preferences, which influences consumer behavior [146]. Dopamine, a neurotransmitter involved in motivation and reward, is hypothesized to control food consumption through regulating its rewarding effects through the *nucleus accumbens* (NA) in laboratory animals [147].

Volkow et al. [147] showed that extracellular dopamine is significantly increased in the dorsal striatum but not in the ventral striatum (the area that included the NA) in response to “non-hedonic” food stimulation, concluding that dopamine is involved in “nonhedonic” food motivation in humans.

Foods may be rejected based on sensory-affective proprieties, attitudes, mentality or symbolic meaning, and ideational factors. A fairly extensive range of functional imagery studies has shown that stimuli that elicit disgust by imposition still capture attention. Therefore, the success of game products could be increased by a scientifically based imposition on the market [148].

Perceptual science occupies a leading position in neuropsychology. Consumer perception is one of the biggest challenges. Since wildlife managers cannot rely only on their own support for hunting activities, it is crucial to investigate the general population perception on wildlife management. For hunting continuity and for a proper utilization of the game reserve, public support is required [143,149]. A choice experiment regarding consumer attitudes toward game meat consumption and attitudes towards hunting activities was carried out by Demartini et al. [33] in Italy. This study revealed that the attitude towards hunting is predominantly positive but with important negative trends (366 positive answers vs. 355 negative answers). Most of the negative attitudes were justified by the fact that a significant proportion of the study participants do not consider hunting as a traditional activity that would provide people with sustainable consumption. The negative attitudes were complemented by the fact that a percentage of participants do not know and are not aware of the role of hunting in reducing overpopulation in game reserves.

Research supports the importance of game meat and the benefits it provides. However, Geisser et al. [48] report that the literature presents a diffuse negative consumer attitude toward hunting. In some developed countries, studies have confirmed that among consumers, the hunting raises ethical, health, and environmental concerns. Although we live in the era of free information, consumers are inclined to show little education about game animals [33,71]. The foundation of a sustainable supply chain depends heavily on perception. Food safety is one of the biggest obstacles to the consumption of wild game meat [150]. In this respect, a study from 2020 by Niewiadomska et al. [38] reported a correlation between a significant consumption of wild game meat and lower safety concerns. The study also concluded that information campaigns directed to diminish consumer safety concerns could help promote the meat’s popularity. Consequently, if attained under correct and normal hunting campaigns, it incorporates several quality characteristics that may appeal to consumers when buying game meat products [71].

Fantechi [150] points out very well the fact that consumer choices should be oriented towards game meat products to be considered an alternative supply chain. The author highlights how important it is to understand the correlation between the consumer attitude towards game meat, which is closely related to different perceptions about hunting.

In order to evaluate the behavior of consumers toward game meat, Table 3 displays some studies randomly selected from the literature for a better view of the rational and emotional reasons and other expressions regarding the behavior of consumers toward game meat. Moreover, in order to evaluate the changes in consumer attitudes over time, consecutive studies from recent years were analyzed.

Surprisingly, the temporal analysis of consumer attitudes towards game meat indicated an important change in a relatively short period of time. In 2020, the arguments for the acceptance of game meat in the diet were mainly due to nutritional benefits, familiarity, and consumption habits [38]. In 2021, in relation to the socio-demographic factors related to education and the professional side, it is highlighted that the arguments for accepting game meat in food are predominantly of a rational nature, centered on the natural content in connection with the quality attributes of the meat [151].

Among the reasons for refusing game meat, both Niewiadomska et al. [151] as well as Czarniecka-Skubina et al. [19] mentioned emotional reasons such as ethical aspects, fear of diseases, method of obtaining, or non-acceptance of the sensory characteristics of game meat. The lack of sufficient information regarding the benefits of game meat and how to prepare it was also mentioned.

Moreover, analyzing the rational side with the emotional side, Niewiadomska et al. [38] stated that rational reasons, compared to emotional ones, have a higher impact on the choice to consume game meat or not. The authors showed that there is an opportunity to elevate the repetitions of game meat consumption, especially in the case of consumers who emphasize concerns related to taste, fat content, nutritional quality, and the origin of meat, by exposing its quality characteristics through producers and distributors.

In association with meat quality attributes, a low connection is observed between qualitative aspects and consumer behavior, most attitudes being generally associated with emotional criteria. However, the studies evaluated are just one example that demonstrates how variable the subject matter is.

Corradini et al. [47] described the socio-demographic variables that have the greatest influence on game meat consumption. The most studied factor was gender, being significant differences in attitude between men and women; men generally have a positive attitude, consuming game meat more often compared to women [72,152]. Table 4 shows the attitude of the game meat literature by age and gender.

Regarding the young people and women, the negative attitudes based on little factual information result in rejecting game meat. In the case of women, emotional reasons may be also considered. An insufficient understanding of the action behind venison induces denial, rejection of healthy products, and stress, while the fear is triggering neurodegeneration. Other parameters that may influence consumer attitudes are represented by age, residence, income, ethnicity, and level of education. In this context, consumers from the rural areas showed a positive attitude, with higher consumption of game meat compared to the inhabitants of the urban area [38,48,155]. In general, a negative attitude towards the perception of game meat and consumption frequency is presented by young consumers [110,152,156], those with low income [71,157] and lower levels of education [38,71,155].

In D’Souza’s work [35], five consumption values were described: functional value, social value, emotional value, an epistemic value and conditional value, values that have been demonstrated during the time to influence consumer decisions. The theory of consumption value (TCV) is considered an essential point of view for the investigation of perceived value by consumers as it helps to predict, describe, and explain choice behavior by focusing on consumption values [158,159,160].

## 4. Conclusions

Game meat is considered a sustainable resource since proper wildlife management offers alternatives to exploit free-ranging animals and providing healthy food in response to global pressures due to the increasing human population. The sensory properties of game meat (color, taste, smell, and aroma) provide a complete perceptual experience. The sensory perception of game meat is responsible for objective and subjective responses that influence consumer behavior by leading to a final decision of acceptance or refusal. The consumer’s final choice is triggered by a number of emotional (ethical concerns, harvesting methods, safety, animal and environmental welfare, familiarity, sensory characteristics) and rational (nutritional value, price, natural origin, place of purchase, process of production) reasons, which can explain the heterogeneity of the population.

Arguments for accepting game meat for consumption are mostly based on rational reasons related to socio-demographic factors (such as gender, age, level of education, and income), centered on quality attributes and superior nutritional and dietetic properties (high protein content, beneficial composition of essential amino acids, limited saturated fat content, high content of polyunsaturated fatty acids), on traditions and consumption habits.

Following the analysis of various scientific sources, we assume that consumer behavior towards game meat is influenced mainly by a misunderstanding of the action behind obtaining it. Misunderstanding and possession of incorrect information are external factors that influence the neurobiological processes through which the unpredictable behavior of the consumer can be explained. However, we believe that the success of venison products is enhanced by a scientifically sound approach.

Prior consumption food is passed through a sensory perception filter. The sensory properties of game meat activate the visual, gustatory, olfactory, and tactile neural sensory system via sensory receptors. The sensory organs transmit sensory stimuli via neural impulses that determine the body’s response to these external factors. The totality of sensations (organoleptic, affective, or related to environmental conditions) caused by different factors interacting continuously produces the complete perceptual experience resulting in the consumer’s final response.

Dopamine, an important neurotransmitter, plays a role in human behavior.

From a research perspective, we propose implementing a methodology for the psycho-sensory evaluation of game meat products by consumers in a welcoming setting, based on a series of scientific data.

## Figures and Tables

**Table 1:** Data about the nutritional value of game meat from different species.

Species	Chemical Composition (%)					Energy (kcal)	Cholesterol (mg/100 g)	Ref.
	Moisture	Dry Matter	Protein	Lipids	Minerals			
Red Deer	76.82 ± 1.16	22.02–23.18	18.71 ± 0.27	2.26 ± 0.03	2.21 ± 0.04	91.04	-	[76]
	76.90	23.10	21.70	0.6	1.11	-	-	[77]
	-	-	22.36	1.90	-	-	70.57	[57]
	75.8	24.2	19.99	3.2	1.2	-	-	[78]
	76.0	24.0	22.2	0.10–0.16	1.32	-	48.6	[79]
	74.16–74.29	25.71 ± 0.13	22.79 ± 0.11	0.50 ± 0.04	1.10 ± 0.03	-	-	[80]
	75.22–77.11	24.78	22.89	0.10–0.96	1.10–1.34	90.0–98.0	45.3–52.8	[58]
	-	-	21.7	2.0	4.55	-	74.0–87.0	[81]
	-	-	22.6	1.2	-	-	59.0	[82]
	75.0	25.0	21.5	0.5–2.0	1.1–1.2	-	-	[83]
	74.1–74.26	25.74–25.90	23.30–24.10	0.25–1.06	1.25–1.34	-	-	[84]
	74.82 ± 0.409	25.17 ± 0.418	22.09 ± 0.356	1.28 ± 0.175	1.03 ± 0.078	158.87 ± 3.38	-	[85]
Fallow deer	74.90	25.10	22.0	2.50	1.08	-	-	[77]
Roe Deer	71.4–74.4	25.6–28.6	22.82–25.70	1.0–2.12	1.29	-	-	[86]
Wild boar	61.83–64.59	35.41–38.17	21.99–22.78	4.52–7.60	-	-	-	[87]
	-	-	22.92	2.82	1.13 ± 0.07	-	70.57–2.49	[57]
	70.50	29.50	21.24–25.87	0.69–2.80	1.03–1.26	101.0–117.0	34.4	[58]
	74.1	25.9	23.75	1.02	1.14	-	-	[78]
	70.5	29.50	25.87	1.55	1.23	-	-	[88]
	74.72	25.28	21.24	2.78	1.23	-	-	[89]
	-	-	21.83 ± 0.57	4.27 ± 1.78	2.91	-	-	[90]
	73.0 ± 0.472	26.99 ± 0.472	21.92 ± 0.273	3.37 ± 0.322	1.11 ± 0.078	159.46 ± 3.58		[85]

**Table 2:** Data about fatty acids and amino acids content of game meat from different species.

Fatty Acids			Amino Acids (mg/100 g)		Ref.
SFA *	MUFA **	PUFA ***	Essential	Non–Essential	
Red Deer					
34.35 ^1^	19.9 ^1^	44.65 ^1^	9504	10,485	[79]
356.0–424.0 ^2^	270.0–372.0 ^2^	259.0–374.0 ^2^	-	-	[81]
53.84 ^1^	26.11 ^1^	20.50 ^1^	-	-	[85]
42.7 ± 2.36 ^1^	22.2 ± 1.43 ^1^	31.0 ± 3.59 ^1^	9590	13,019	[80]
30.4–38.2 ^1^	15.3–22.7 ^1^	37.6–50.1 ^1^	-	-	[58]
42.13 ^1^	26.57 ^1^	23.47 ^1^	-	-	[57]
42.13	26.56	23.38			[100]
0.17 ^3^	0.07 ^3^	0.01 ^3^	-	-	[101]
Wild Boar					
35.79 ^1^	45.29 ^1^	18.91 ^1^	9797	11,875	[85]
31.6–44.7 ^1^	30.2–46.8 ^1^	17.3–30.5 ^1^	-	-	[58]
34.79 ^1^	35.63 ^1^	17.25 ^1^	-	-	[100]
35.25 ^1^	42.74 ^1^	20.15 ^1^	-	-	[102]
35.40 ^1^	48.05 ^1^	16.55 ^1^	-	-	[89]
36.74 ^1^	33.20 ^1^	30.06 ^1^	-	-	[90]
32.67–34.28 ^1^	41.79–44.31 ^1^	17.12–19.19 ^1^			[87]
Roe Deer					
40.90–42.13	21.10–26.56	23.48–37.70			[100]
1.17^3^	0.66 ^3^	0.11 ^3^	-	-	[101]

^1^ g/100 g of total fatty acids; ^2^ g/kg of total fatty acids; ^3^ g/100 g of game meat; * SFA—saturated fatty acids (C14:0–C22:0); ** MUFA—monounsaturated fatty acids (C16:1 n-9–C22:1 n-9); *** PUFA—polyunsaturated fatty acids (C18:2 n-6; C18:3 n-3, n-6; C20:2; C20:3 n-3, n-6; C20:4 n-6; C20:5 n-3; C22:4 n-6; C22:5 n-3, n-6; C22:6 n-3).

**Table 3:** Expressions used in studies to determine consumers’ eating habits regarding game meat.

Socio-Demographic Characteristics			Statements of Acceptance	Statements of Refusal	Ref.
2020					
N = 450	Gender	41.3% Female; 58.7% Male	Nutritional and health value Weight control Low fat content Sensory appeal Natural content Familiarity	Convenience (purchase and preparation methods) Safety concerns Fear of an unknown product Ethical concerns (production methods, environment and animal welfare) Price	[38]
	Education	2.4% Primary; 77.3% Secondary; 20.3% Higher			
	Age (years)	25–34 (37.1%); 35–44 (27.1%); 45–54 (24.7%); 55 and over (11.1%)			
	Place of living	54.7% Town; 45.3% Village			
2021					
N = 450	Gender	41.3% Female; 58.7% Male	Quality attributes Natural content Price (paying for better quality) Purchase and preparation method	Obtaining method Health risks (infection with parasites/zoonoses; increase in cholesterol level, weight control, risks of contamination with different xenobiotics) Not enough information (culinary programs or books, producers websites, nutrition specialists, advertising) Familiarity	[145]
	Education	2.4% Primary; 77.3% Secondary; 20.3% Higher			
	Age (years)	25–34 (37.1%); 35–44 (27.1%); 45–54 (24.7%); 55 and over (11.1%)			
	Place of living	54,7% Urban area; 45.3% Rural area			
	Professional situation (work)	75.6% full-time; 6.4% part-time; 10.7% partner; 3.3% study; 4.0% pensioner			
2022					
N = 1251	Gender	52.2 Female; 47.8% Male	Distinctive taste of game meat Health properties Family traditions Participation in hunting Availability of game meat Popularity of this game	High price Low availability Unacceptable sensorial appeals Not enough information about health benefits Ethical aspects Fear of disease No family tradition	[19]
	Age (years)	18–30 (39.5%); 31–40 (20.2%); 41–50 (17.8%); 51 and over (22.5%)			
	Education	3% Vocational or primary; 36.4% Secondary; 60.6% Higher			
	Place of living	33,8% Rural area; 66.2% Urban area			
	Financial situation	18% Very Good; 56.2% Good; 24.5% Not good, not bad; 1.3% Bad and very bad			
	Game consumers	26.2% Hunters; 73.8% Others			
	Hunters (hunting years)	0–5 (21.10%); 6–10 (22.6%); 11 or over (56.3%)			

**Table 4:** The reasons for the attitude of game meat consumers according to the socio-demographic variable.

Socio-Demographic Variables		Attitudes	Reasons	Ref.
Young		Negative	More information needed; Territorial influences; In progress or completed studies	[38,71,153,154]
Gender	70.7% Female; 29.3% Male			
Age (years)	Below 20 (20.4%); 20–29 (47.2%); 30–39 (12.4%)			
Men		Positive	Passion for hunting led them to inform themselves; The perception of the unique taste	
Women		Negative	More scientific information needed.	

## Data Availability

Data sharing is not applicable to this article.
